# Protecting, promoting and supporting breastfeeding in all policies: reframing the narrative

**DOI:** 10.3389/fpubh.2023.1149384

**Published:** 2023-06-16

**Authors:** Cecília Tomori

**Affiliations:** Department of Population, Family and Reproductive Health, Johns Hopkins University School of Nursing and The Johns Hopkins Bloomberg School of Public Health, Johns Hopkins University, Baltimore, MD, United States

**Keywords:** breastfeeding, food security, health security, climate change, infant and young child feeding in emergencies (IYCF-E), disasters, health policy, commercial milk formula marketing

## Abstract

Recent research highlights the importance of breastfeeding to health across the lifecourse, yet inadequate investment to facilitate breastfeeding according to World Health Organization recommendations threatens to undermine breastfeeding’s protective effects. Western media narratives often fail to convey the significance of breastfeeding, and such narratives can hinder efforts to direct sufficient resources to scaling up effective systems and generating policy change. Delayed action disproportionately harms poor and marginalized communities. The urgency of making these investments in an era of rapidly intensifying climate change and other crises is clear. Reframing the narrative is needed to better appreciate the significance of breastfeeding as well as to recognize and address extensive efforts of undermine it. Evidence-based scientific, health professional and media discussions are necessary to recognize breastfeeding as foundational to food and health security and to enact change so that protecting, promoting and supporting breastfeeding is integrated across all policies.

## Introduction

Contemporary Western debates frequently frame breastfeeding in the context of individual, highly moralized decisions (1, 2). While similar tropes may be increasingly common elsewhere, narrative frameworks in Western settings have an outsize influence because these wealthy nations assert considerable power in policy decisions that allocate global funding resources as well as over the regulatory environment that shapes infant feeding decisions. These moralized media debates, however, are often misguided and do not reflect the everyday realities of breastfeeding decisions. Nearly half of families globally live in poverty (3), and continue to experience inadequate structural and interpersonal support pre-and postnatally for breastfeeding (4, 5) while simultaneously subjected to aggressive, predatory marketing of the commercial milk formula (CMF) industry (6). Models estimate that over 800,000 infants and young children die each year due to not being appropriately breastfed, and over 100,000 mothers annually die of ovarian and breast cancers, and experience substantial additional morbidity due to suboptimal breastfeeding (7, 8). While the toll of suboptimal breastfeeding affects populations across high, middle, and low-income settings, poor and marginalized populations are disproportionately harmed by the inadequacy of supports for breastfeeding; they bear most of the burden of infant and maternal death and long-term health consequences (5, 7). In a time of increasing inequality and rapidly accelerating crises (9–12), a significant reorientation of media narratives is necessary to reframe the protection, promotion and support of breastfeeding as foundational to food and health security (13, 14) and as a policy priority across multiple sectors ranging from food and health systems, to emergency preparedness and climate mitigation. This warrants significant scale-up of national and international investment.

### Breastfeeding’s evolutionary roots and contemporary public health significance

Recent research has shown that the significance and scope of impacts of breastfeeding on health and human development is far greater than even previously recognized (5). These impacts are grounded in evolutionary adaptations that underlie lactation, some of which predate mammals (15). Contemporary human lactation adaptations reflect specific mammalian reproductive and infant care strategies that are characteristic of primate species but also have unique elements because of our own human evolutionary history. All primates give birth to large-brained infants that require close contact, frequent nursing, and an extended period of postnatal care. Humans, however, are born in an exceptionally immature state and have an even longer postnatal period of brain maturation that requires intensive bodily contact and caregiving (16, 17). Humans have a series of biocultural adaptations to accommodate these needs, including multiple caregivers in the community, some of whom may also serve as alloparents and may also engage in cooperative lactation practices (18). The biological adaptations within this lactation system are far reaching—ranging from cardiac, thermoregulatory, metabolic, circadian, and psychosocial co-regulation to a sophisticated system of immunological communication within which the infant’s immune system matures over time (5). These adaptations have played—and continue to play—a crucial role in ensuring survival and facilitating development in a vast array of environments in which humans live. Public health research demonstrates the far-reaching effects of breastfeeding in providing protection from malnutrition, dehydration, infectious-and non-communicable diseases as well as in shaping cognition and development (5).

### Historical decline in breastfeeding

As a biocultural process, successful lactation relies on an enabling social environment that is a product of a number of intersecting forces. While we know that breastfeeding has deep evolutionary roots and has been the historical norm across populations, its practice and duration have varied across populations (19). The greatest shift in infant feeding practices, however, came about relatively recently as a part of enormous social changes driven by the rise of colonialism and racial capitalism, and accompanying medicalization (15). Racial capitalism, as introduced by Robinson, refers to the inextricably linked systems of the accumulation of wealth in capitalism and racial oppression and exploitation (2). These violent, extractive systems caused profound disruptions to entire lifeways across the globe. They uprooted Indigenous systems of knowledge and increasingly replaced them with biomedicalized systems rooted in Western, white, elite cultural assumptions of infant care. These systems aimed to regulate women’s productive and reproductive capacities and regiment infant care, including feeding and sleep (15). For instance, in the 1930s Belgian colonizers were concerned with shortening the duration of lactation and reducing responsive breastfeeding in the Congo because they viewed these practices as “uncivilized” and because they wanted to reduce birth spacing and increase fertility to be able to extract more labor from the colony (20). Coupled with increasingly aggressive efforts to market commercial milk formulas (CMF), and the consolidation of medical authority in medically-supervised births, there was a profound decline in breastfeeding that reached its nadir in the middle of the 20th century. While global efforts in the second half of the 20th century have rallied in advocacy and support for breastfeeding within global public health and biomedical guidance, material investment in structural changes has lagged behind and continues to threaten breastfeeding globally (5).

### The historical role of the CMF industry in undermining breastfeeding

The CMF industry has played a powerful role in shaping nutritional science and policies. From its origins in the 19th century, the industry preyed on existing cultural concerns about infant feeding and the rapidly changing social circumstances that families faced during the acceleration of industrialization, urban migration, and increasingly challenging labor conditions (2, 21, 22). Additionally, CMF marketing tactics drew on the scientific authority of male health professionals and scientists who argued that these milk products solve concerns about the adequacy of breastfeeding and breastmilk and were superior to breastfeeding. These arguments were aggressively pursued in marketing campaigns in Europe. Increasingly, however, these products were incorporated into colonial and later post-colonial economic systems (20, 21, 23, 24). In the 1950s Nestlé, for instance, aggressively promoted formula as a solution to address malnutrition across Africa, particularly through establishing relationships with colonial medical professionals as well as NGOs. These efforts paid off handsomely for the company as it shaped the malnutrition research agenda and exploited it as part of its marketing efforts (24, 25). The distribution of CMF became part of the course for governmental public health efforts that aimed to “improve” nutrition, particularly for Indigenous populations. The Canadian government, for instance, targeted Indigenous breastfeeding practices within public health systems and instructed scheduled feedings with CMF (26). These efforts were part of large-scale settler colonial efforts to undermine and erase Indigenous cultural and childcare practices, which included the forced removal of children to residential schools (26). Only after the devastating increases in mortality were documented did the government reverse course (26).

Some of the most aggressive tactics in poorer settings later gained media attention, galvanized by physicians, nutritionists, and community activists who witnessed severe illness and consequent death (2, 21, 27). The scandals were prompted by aggressive marketing tactics, including egregious examples of health professionals, and even sales representatives dressed as health professionals, providing formula samples and extolling the virtues of CMF to new mothers in maternity wards (2). The immediate impacts were visible and large scale, crystallized in The Baby Killer report published in 1974 (21). Observers described bottle-fed babies sickened primarily from contaminated water, lack of infrastructure to keep bottles clean, the dilution of CMF when money ran short, and sickness and death due to lack of protection against infectious disease conferred by breastfeeding. The devastating impacts of these tactics spurred global outrage, led to the Nestlé boycott, and ultimately to laying the foundations of contemporary global health efforts to protect, promote, and support breastfeeding and regulate CMF marketing via the World Health Assembly’s adoption of the of the International Code of Breast-milk Substitutes (the Code) in 1981 (2, 21).

### Continued challenges in addressing the influence of CMF industry marketing efforts

Over the past four decades, despite continued efforts and subsequence resolutions to the Code few settings have implemented strong protections for breastfeeding (6). While measures for the timely initiation of breastfeeding, exclusivity, and duration reflect progress in many areas of the world, marketing and corporate political activity have also accelerated, raising more profits than ever before (28). The CMF industry is now a USD 55 billion business and has become a powerful player in every realm from scientific and health professional communities to public-private partnerships in nutrition, to policy mechanisms in global economic and trade policies that enables its virtually unchecked growth (28, 29). Self-reported insufficient milk (SRIM) is a key reason why mothers introduce CMF and stop breastfeeding sooner than they desire, and half of breastfeeding mothers report SRIM (5). Although there are multiple inadequacies in the health system and workplace protections, which also influence these outcomes, marketing is a key driver (6).

A common strategy employed in CMF marketing is to reframe normal human infant behavior as problematic, and then position CMF as the solution to this problem. For instance, CMF advertisements commonly refer to addressing crying and fussiness, and improving sleep (6). These are typical infant behaviors that require responsive care—not CMF (5). Introducing CMF undermines exclusive breastfeeding and displaces opportunities for breastfeeding and therefore stimulating milk production, leading to premature breastfeeding cessation (5).

Recently, the science on human milk has received considerable industry attention and funding. This work focuses on the components of milk that can be added to CMF (30–32). The addition of these components is then marketed to parents to claim that the product is “more similar” to human milk and will produce healthier and more intelligent infants and children—even in the absence of scientific support for these claims (6). The reductive, component-based approaches to human milk abstract away the complex dynamics and variability of the living substance of human milk and the breastfeeding process itself. Human milk reflects a wealth of evolutionary adaptations that have shaped human milk in the context of breastfeeding—originating out of the bodily proximity of infants and their mothers and in response to the broader biosocial environment (19, 33). Efforts to extract human milk and its components as *products* out of the *process* of breastfeeding reflect tactics built on the foundations of commercial exploitation of infant feeding (1, 15, 34). These tactics rely on the separation of product from process to ultimately displace breastfeeding and replace it with CMF.

Importantly, few are aware of the range and extent of industry tactics to undermine breastfeeding and expand their markets. This includes the scientific and health professional community who is subject to industry framings of science and health professional education (6)—all of which become part of the unconscious background for providing guidance and recommendations for infant feeding. For instance, health professional education about breastfeeding may be provided by the CMF industry or its front groups, and the CMF industry frequently sponsors health professional associations, conferences, as well as scientific research (6). At the same time, governments have not provided adequate resources for independent, evidence-based lactation training for health professionals or sufficient investment in research. This leaves health professionals vulnerable to industry influence, and in turn shapes their advice to families. Specialty CMF, such as products claiming to address allergy, for instance, have been particularly successfully marketed to health professionals who, in turn, recommend them to parents (6).

The downplaying of the significance of lactation in human health paired with what may be perceived as scientific authority on the replaceability of breastfeeding is also a powerful tool for persuading policy makers that there is little need for marketing or additional safety regulations (2). This means that industry tactics fade into the cultural background for policy makers who may inadvertently replicate ethnocentric assumptions about lactation and further corporate agendas. This is particularly dangerous because policy change is unlikely in the absence of broader awareness.

As one example, the US medical community has been slow to fully support breastfeeding and the leading pediatric association has an ongoing philanthropy that is partially funded by CMF manufacturers (2). The 2-year recommended duration for breastfeeding per World Health Organization (WHO) guidance was only adopted in US pediatric guidelines in 2022 (1, 2, 35). Even then, the guidance was controversial. It came amid a national CMF crisis that was prompted by the shutdown of a plant that produced a large portion of CMF in the US and was linked to contaminated CMF that sickened and killed some infants (2). For many, the pediatric guidance furthered a sense that agencies expect birthing people to take on even more bodily labor for lactation without any additional support. The US stands out among wealthy nations for its lack of federal paid leave, poor workplace protections, and highly fragmented access to healthcare, which also limits access to skilled lactation support in perinatal care and in the community (36, 37). At the same time, the US has also been a site of aggressive marketing efforts to consumers and health professionals, and industry efforts to capture the regulatory environment which facilitates these efforts (2, 37). The formula crisis has prompted greater media attention to industry efforts to hinder adoption of stronger regulations (2), but there is still limited awareness of broader industry efforts to sow doubt about scientific evidence on breastfeeding, undermine structural support for lactation (e.g., paid leave and regulatory protections) and shape public norms and narratives about breastfeeding (6). Media debates that focus on individualized responsibility around breastfeeding can serve as a diversionary tactic away from corporate responsibility and the need for policy action to create supportive systems and regulations on corporate activity (38).

### The urgency of integrating lactation in emergency preparedness and climate change policies

Insufficient appreciation of lactation’s enormous role in securing health is reflected in inadequate integration of lactation across public health domains. For instance, few nations integrated appropriate guidance on breastfeeding into their COVID-19 responses, often leading to maternal-infant separation policies that had a wide range of negative consequences, including on breastfeeding (39). Similarly, protecting promoting and supporting lactation is insufficiently integrated into disaster preparedness and climate mitigation policies. Yet infant and young child mortality is highest in the wake of both slow and fast disasters, which are rapidly accelerating with climate change (40–42). In these settings, it’s particularly essential to provide appropriate support for continued breastfeeding, and to avoid unnecessary distribution of CMF, which undermines breastfeeding.

The most recent IPCC report and the Lancet commission (43, 44) both pointed out that the world is going to experience more extreme weather, including storms and drought, greater heat stress, and mass displacement of people due to increasingly hostile conditions that endanger life and limit the ability to grow food and access water. Moreover, further pandemics will be fueled by the expansion of vector-born disease and habitat encroachment. These effects already disproportionately affect the world’s poorest, predominately located in the Global South and poor and marginalized people in the Global North (44, 45). Even in comparatively wealthier settings, each time there are strong storms, flooding, electrical outages, and water shortages or water safety concerns—infants reliant on CMF are at immediate heightened risk for dehydration, hunger, and diarrheal disease (46–48). Inadequate attention to these critical issues means a failure to invest adequate resources into the everyday task of creating an enabling environment for breastfeeding to prevent accelerating harms.

## Discussion: enacting change

Enacting change to fully recognize breastfeeding as foundational to health, as well as food and health security, requires simultaneously addressing the chronic underinvestment in creating enabling environments and emergency preparedness and mitigation policies. In some cases, national recommendations may align with World Health Organization recommendations. However, stated support without adequate investment is vastly inadequate to create an environment that makes these recommendations possible.

Investing in health professional training and in health systems that support birthing people is needed so that they are adequately prepared to support lactation and they can implement best practices without industry interference (4). Similarly, paid leave and workplace protections are crucial to be able to continue breastfeeding once it is established (49). Additionally, regulatory reform is necessary to limit the influence of the CMF industry across multiple areas of policy and sectors (28). Investment in multi-sectorial efforts to address everyday challenges have an impact that stretch beyond these scenarios when emergencies strike (4). The more families and communities are supported in breastfeeding practices, the more likely that their infants will survive emergency scenarios and the pressures imposed by climate change and continue to thrive. Furthermore, the more support lactation receives in the perinatal period and early childhood, the better longer-term outcomes for morbidity and mortality. These supports are also pivotal in efforts to address health inequities within settings. The physical, mental health and financial impacts of these conditions are far greater than the investment required to prevent them (8, 50).

To create momentum for policy change across multiple sectors, public health and healthcare professionals, scientists, and journalists require training in recognizing and addressing the industry playbook that has been deployed to undermine breastfeeding – as well as other areas of public health (51, 52). Scholars and practitioners need to learn that industry tactics are far-reaching and assert influence not only over lactation but all other areas of health, that are explored under the umbrella of the emerging field of commercial determinants of health (38, 51, 53). This training is essential to help create evidence-based framing of discussions of breastfeeding and lactation. Creating scientific, health professional and media environments that present a more accurate, and nuanced understanding of lactation can greatly facilitate societal investment needed to create an enabling environment.

## Conclusion

The impact of breastfeeding on the health of infants and young children, their mothers and birthing people, and entire communities is unparalleled (5). Significant investment and education is necessary to scale up investment and support to enable breastfeeding across sectors (5). We must continue to reframe messages around lactation and facilitate conversations to enhance public, scientific, health professional, and policy makers’ understanding of the value of lactation as securing food, hydration, and ultimately survival and long-term health.

## Data availability statement

The original contributions presented in the study are included in the article/supplementary material, further inquiries can be directed to the corresponding author.

## Author contributions

The author confirms being the sole contributor of this work and has approved it for publication.

## Conflict of interest

The author declares that the research was conducted in the absence of any commercial or financial relationships that could be construed as a potential conflict of interest.

## Publisher’s note

All claims expressed in this article are solely those of the authors and do not necessarily represent those of their affiliated organizations, or those of the publisher, the editors and the reviewers. Any product that may be evaluated in this article, or claim that may be made by its manufacturer, is not guaranteed or endorsed by the publisher.
